# Supplementary material for: Enhanced Antibacterial Activity of a Cationic Macromolecule by Its Complexation with a Weakly Active Pyrazole Derivative
Source: Biomedicines. 2022 Jul 6;10(7):1607. doi: 10.3390/biomedicines10071607 (PMC9313313; doi:10.3390/biomedicines10071607)
Supplement: Supplementary file 1 [file biomedicines-10-01607-s001.zip › biomedicines-1761528-supplementary.pdf]

Supplementary Materials

Enhanced Antibacterial Activity of a Cationic Macromolecule by its Complexation with a Weakly Active Pyrazole Derivative

Anna Maria Schito [1] Debora Caviglia [1,*], Chiara Brullo [2], Alessia Zorzoli [3], Danilo Marimpietri [3] and Silvana Alfei [2,*]

[1] Department of Surgical Sciences and Integrated Diagnostics (DISC), University of Genoa, Viale Benedetto XV, 6, 16132 Genoa, Italy; amschito@unige.it (A.M.S.)
[2] Department of Pharmacy (DIFAR), University of Genoa, Viale Cembrano, 16148 Genoa, Italy; chiara.brullo@unige.it (C.B.)
[3] Cell Factory, IRCCS Istituto Giannina Gaslini, via Gerolamo Gaslini 5, 16147 Genoa, Italy; alessiazorzoli@gaslini.org (A.Z.), danilomarimpietri@gaslini.org (D.M.)
*Correspondence: alfei@difar.unige.it; Tel.: +39 010 355 2296 (S.A.); debora.caviglia@edu.unige.it (D.C)

Table S1. Main physicochemical properties of CB1H-P7 NPs.

<table>
<tr><th>Analysis</th><th colspan="2">CB1H-P7 NPs</th></tr>
<tr><td>FTIR [cm $^{-1}$]</td><td colspan="2">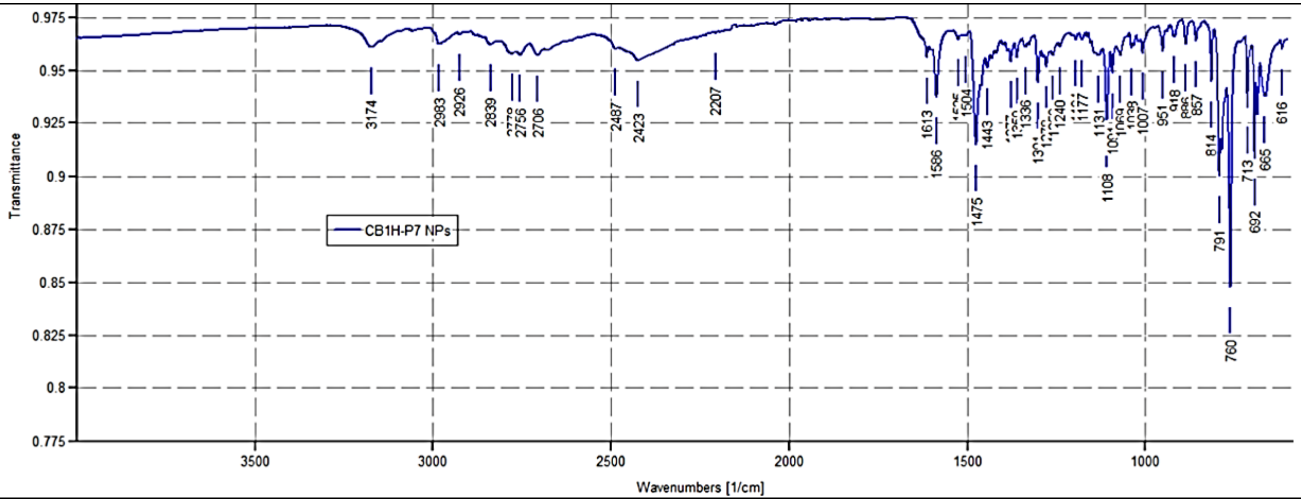
</td></tr>
<tr><td>Principal Components Analysis (PCA)</td><td colspan="2">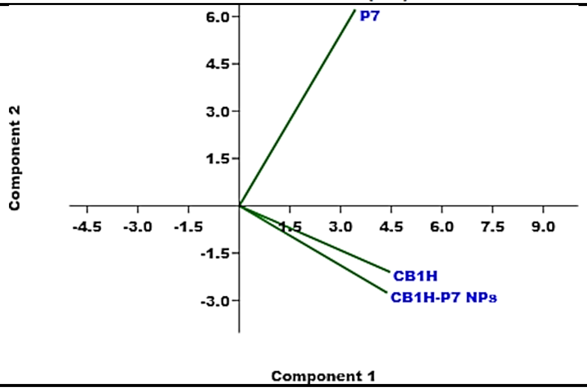
</td></tr>
<tr><td>UV-Vis</td><td>Ultraviolet Spectrum</td><td>λ_{abs} = 250 nm</td></tr>
<tr><td rowspan="2">UV-Vis</td><td>DL (%)</td><td>48.6±1.4</td></tr>
<tr><td>EE (%)</td><td>81.5±2.50</td></tr>
<tr><td>DL% (UV-Vis)</td><td>MW</td><td>26,623.9±260.3</td></tr>
</table>

Scanning Electron Microscopy (SEM)		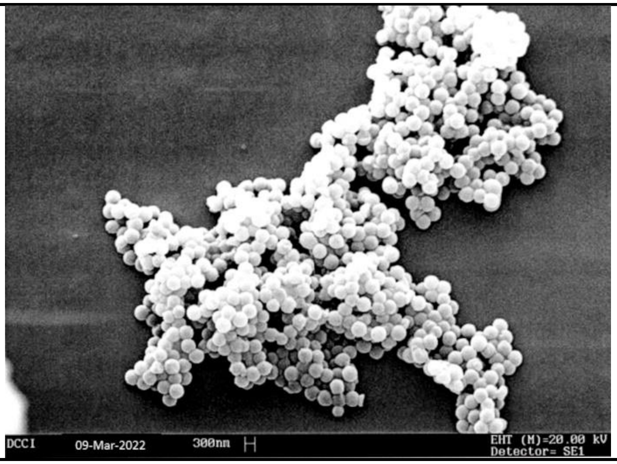	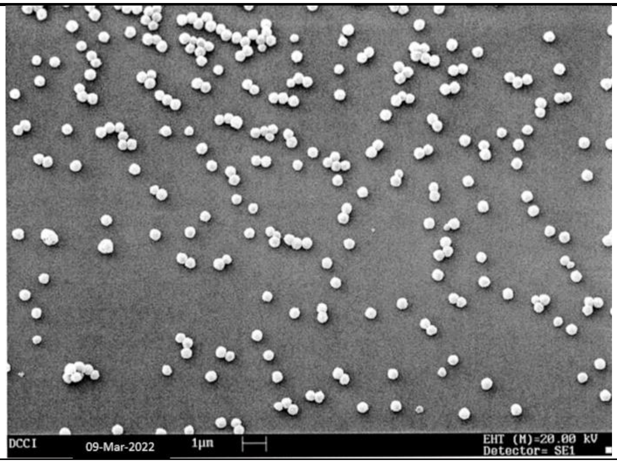
		P7 (342.9±57.2 nm)	CB1H-P7 NPs (332.8±67.9 nm)
DLS [1] Analysis	Z-Ave [2] (nm)<br>PDI [3]	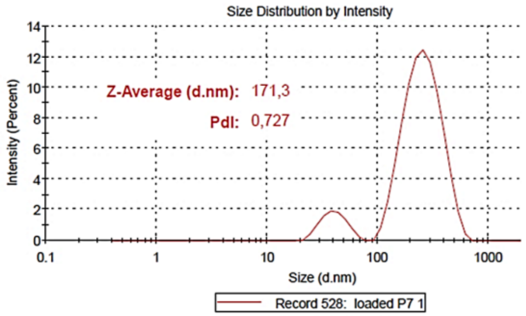	142.9±20.1<br>0.626±0.071
	ζ-p [4] (mV)	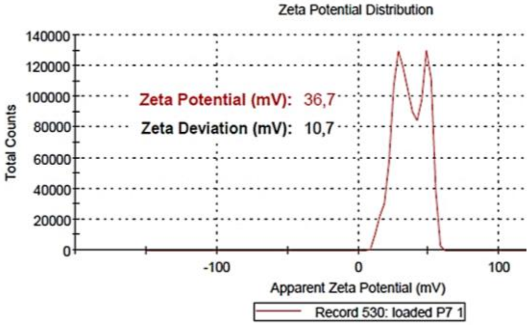	+36.7±10.7

Dialysis Method (UV-Vis)	Cumulative Release (%, 24h)	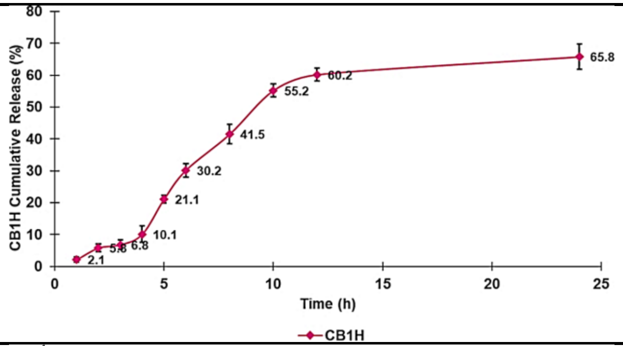
	Mathematical Model	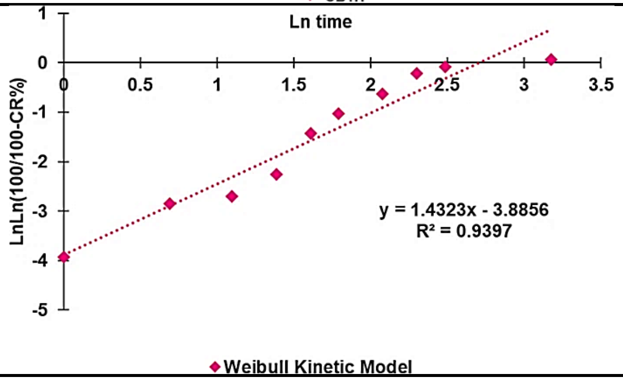
	Mechanism	(β>1) Complex Mechanisms
Potentiometric Titration#	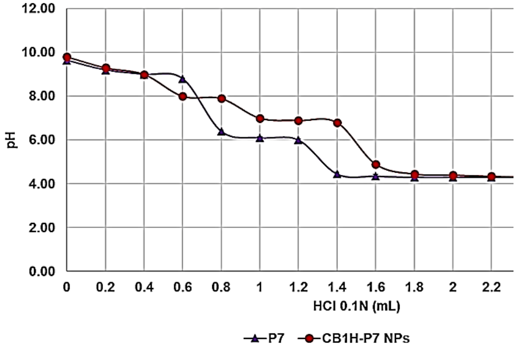 Potentiometric Tritration	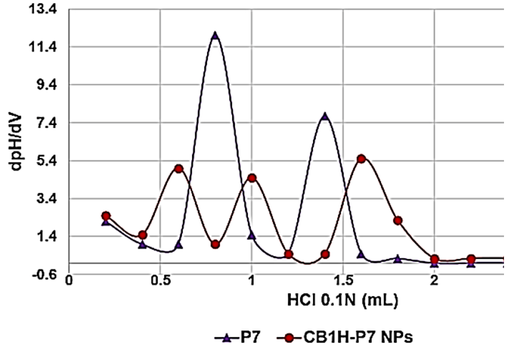 First Derivative

[1] dynamic light scattering; [2] hydrodynamic diameters of particles; [3] polydispersity indices; [4] measures of the electrical charge of particles suspended in the liquid of acquisition (water).
